# Immunization With a DNA Vaccine Encoding the *Toxoplasma gondii*’ s GRA39 Prolongs Survival and Reduce Brain Cyst Formation in a Murine Model

**DOI:** 10.3389/fmicb.2021.630682

**Published:** 2021-04-28

**Authors:** Yuchao Zhu, Yanan Xu, Lu Hong, Chunxue Zhou, Jia Chen

**Affiliations:** ^1^Department of Radiology, The Affiliated Hospital of Medical School of Ningbo University, Ningbo, China; ^2^The Ningbo Women and Children’s Hospital, Ningbo, China; ^3^Department of Pathogen Biology, School of Basic Medical Sciences, Cheeloo College of Medicine, Shandong University, Jinan, China

**Keywords:** *Toxoplasma gondii*, GRA39, DNA vaccine, toxoplasmosis, Th1

## Abstract

*Toxoplasma gondii*, an obligate intracellular protozoan parasite, can cause infect almost all warm-blooded animals and humans. To evaluate the immunogenicity and protective efficacy of *T. gondii* GRA39 (TgGRA39) in mice by using DNA immunization, we constructed a recombinant eukaryotic plasmid pVAX-TgGRA39. The specific immune responses in immunized mice were analyzed by serum antibody and cytokine measurements, lymphocyte proliferation assays and flow cytometry of T lymphocyte subclasses. Also, protective efficacy against acute and chronic *T. gondii* infection was assessed by observing the survival time after challenge with the highly virulent *T. gondii* RH strain (Genotype I) and counting the number of cyst-forming in brain at 4 weeks post-infection with the cyst-forming PRU strain of *T. gondii* (Genotype II), respectively. Our results showed that DNA immunization with pVAX-GRA39 via intramuscular injection three times, at 2-week intervals could elicit humoral and cellular immune response, indicated by enhanced levels of IgG and IgG2a antibodies (a slightly elevated IgG2a to IgG1 ratio), and increased levels of cytokines IFN-γ, IL-2, IL-12, IL-17A, IL-17F, IL-22 and IL-23 and percentages of CD3+ CD4+ CD8- and CD3+ CD8+ CD4– T cells, in contrast to non-immunized mice. The significant increase in the expression levels of IL-6, TGF-β1, IL-1β, and the transcription factor factors RORγt, RORα, and STAT3 involved in the activation and pathway of Th17 and Tc17 cells, were also observed. However, no significant difference was detected in level of IL-4 and IL-10 (*p* > 0.05). These effective immune responses had mounted protective immunity against *T. gondii* infection, with a prolonged survival time (16.80 ± 3.50 days) and reduced cyst numbers (44.5%) in comparison to the control mice. Our data indicated that pVAX-TgGRA39 could induce effective humoral, and Th1-type, Th17, and Tc17 cellular immune responses, and may represent a promising vaccine candidate against both acute and chronic *T. gondii* infection.

## Introduction

*Toxoplasma gondii* is an obligate intracellular protozoan parasite that infects a broad range of warm-blooded vertebrates, including humans and birds (Gangneux and Dardé, 2012; Hakimi et al., 2017; Kochanowsky and Koshy, 2018). As a ubiquitous human and veterinary pathogen, it has a global distribution, and it is estimated that 30% of the global human population may be infected (Wang et al., 2017d). *T. gondii* infection is usually asymptomatic or subclinical in immunocompetent individuals, while in immunocompromised individuals (e.g., AIDS, patients receiving organ transplants or undergoing cancer treatment, developing fetuses), severe and potentially lethal toxoplasmosis may be developed (Petersen et al., 2012; Wang et al., 2017c; Wohlfert et al., 2017). *T. gondii* infection in animals can cause abortion and neonatal death, particularly in sheep and goats, resulting in significant economic losses to animal production (Tenter et al., 2000).

Despite that some drugs could be used to treat acute *T. gondii* infections, no available drugs can eliminate the parasite cysts effectively (Wang et al., 2019). Immunoprophylaxis with effective vaccines would be of high priority to control *T. gondii* infection (Zhang et al., 2013, 2015a). Although a commercially licensed live-attenuated vaccine (Toxovax based on the S48 strain) is available for the veterinary industry in some countries, it was not used for humans and food-producing animals due to its side effects, including virulence reversion, as well as the potential pathogenicity in immune-compromised individuals, and inadequate efficacy (Jongert et al., 2009). Thus, it is imperative to develop safe, practical and effective vaccines against *T. gondii* infections in humans and animals.

DNA-based vaccines offer an alternative approach for immunization, with the ability of eliciting effective humoral and cell-mediated immunity against cellular pathogen invasions in animal models by an unique way of delivering the expressed protein as an endogenous antigen (Liu, 2011). In the past years, a number of antigens has been identified as DNA vaccine candidates, which mainly focused on some virulence factors of *T. gondii* (Li and Zhou, 2018). However, no any identified DNA vaccine candidates can induce complete protective immunity against *T. gondii* infection, so the screening of novel potential vaccine candidates against *T. gondii* infection will be important for DNA vaccination (Zhang et al., 2013; Li and Zhou, 2018). Thus, current studies of *T. gondii* vaccines have been focused on finding novel vaccine candidates and evaluating their protective immunity against toxoplasmosis in animal models.

The dense granule protein 39 (GRA39) was recently identified as a novel *T. gondii* virulence factor, which is critical for efficient replication within its host cell and plays a key role in pathogenesis (Nadipuram et al., 2016). Due to the critical biological roles of GRA39 in *T. gondii*, it could represent a potential vaccine candidate against *T. gondii* infection. However, no studies have evaluated the vaccine potentiality of GRA39 gene. Thus, the objective of this study was to determine the immunogenicity of TgGRA39 in a murine model and to assess protective effects of this DNA vaccine candidate.

## Materials and Methods

### Mice and Parasite

Specific-pathogen-free (SPF) female Kunming mice of six to eight weeks old were purchased from Zhejiang Laboratory Animal Center, Hangzhou, China, which have been used as a proper model for *T. gondii* vaccine evaluation and challenge in previous studies (Chen et al., 2016; Zhang et al., 2018a; Zhu et al., 2020). All mice were maintained in strict accordance according to the Animal Ethics Procedures and Guidelines of the People’s Republic of China. Animal experiments were approved by the ethical committee of Ningbo University [permission: SYXK(ZHE)2013-0190].

Tachyzoites of the highly virulent RH strain of *T. gondii* (Type I) and the brain cyst-forming of the PRU strain (Type II) were used for the *in vivo* challenge of mice. These two strains of *T. gondii* were propagated and harvested as described in our previous studies (Chen et al., 2016; Zhang et al., 2018a; Zhu et al., 2020). The obtained tachyzoites were also used for total RNA extraction (RNAprep Pure Tissue Kit, Sangon, China) and the preparation of *T. gondii* lysate antigen (TLA), as previously described (Chen et al., 2015).

### Epitope Prediction

DNASTAR software (Madison, WI, United States) and its program of “Editseq” and “Protean” was operated and used to predict epitope and analyze the immunogenicity of *T. gondii* GRA39, including its antigenic index, hydrophilicity, flexible regions, and surface probability.

### Construction of the Eukaryotic Expression Plasmid

The coding sequence of the TgGRA39 gene (ToxoDB: 289380\033840) was amplified by PCR from *T. gondii* strain RH DNA for constructing the pVAX-GRA39 plasmid, with a pair of oligonucleotide primers (forward primer, 5′-CGGGGTACCCCGATGGGGCACCCTACCTCTTTC-3′, and reverse primer, 5′-TGCTCTAGAGCATCACGTTTCCGGTGGTGGC-3′), and *Kpn*I and *Xba*I restriction sites were introduced. The obtained PCR product was ligated into the pMD-18 T Vector (TaKaRa, China), generating pMD-GRA39. The GRA39 fragment was cleaved by *Kpn*I/*Xba*I from pMD-GRA39 and then subcloned into pVAX I (Invitrogen, United States) which were cleaved by *Kpn*I/*Xba*I, then generated the plasmid pVAX-TgGRA39 using T4 DNA ligase. The recombinant plasmids were identified by PCR, double restriction enzyme digestion and sequencing. DNASTAR software (Madison, WI, United States) was used to predict the potential epitopes and to analyze the biochemical indexes of GRA39, such as antigenic index, hydrophilicity, as well as flexible regions, and surface probability.

The positive plasmids were purified from transformed *Escherichia coli* DH5α cells by anion exchange chromatography (EndoFree plasmid giga kit, Qiagen Sciences, MD, United States) according to the manufacturer’s instructions. The concentrations of plasmids were determined by spectrophotometer at OD260 and OD280, and then dissolved in sterile phosphate-buffered saline (PBS) with a final concentration of 1 mg/mL and stored at −20°C until use.

### Expression of pVAX-GRA39 Plasmid *in vitro*

The Lipofectamine 2000 reagent (Invitrogen) was used for transfection of pVAX-GRA39 into Marc-145 cells, according to the manufacturer’s instructions, as described previously (Chen et al., 2016). In brief, 48 h post-transfection, the cells were fixed with cool acetone for 30 min and expression of pVAX-GRA39 was examined using the indirect immunofluorescence assay (IFA) followed by anti-*T. gondii* polyclonal antiserum (1:50) and a FITC-labeled donkey-anti-goat IgG (Proteintech Group Inc., Chicago, IL, United States; 1:1000). The monolayers binding marker were covered with glycerine and examined for specific fluorescence under a Zeiss Axioplan fluorescence microscope (Carl Zeiss, Germany). Marc-145 cells transfected with empty pVAX I were used as the negative control.

Expression of pVAX-GRA39 in the transfected cells was then examined by western blotting. Briefly, the Marc-145 cells transfected with pVAX-GRA39 were lysed by freezing and thawing for five times, and then the lysates were subjected to SDS-PAGE. Thereafter, the nitrocellulose (NC) membrane (Sigma, United States) was incubated with 5% bovine serum albumin (BSA) in PBST (PBS with 0.05% Tween-20) at room temperature (RT) for 1 h to block the non-specific binding sites followed by electrotransfer. After washing for three times with PBST, the membrane was cultured with pVAX-GRA39-vaccinated mouse sera (diluted in 1:500) at RT for 1 h, which was collected at 2 weeks after the third immunization. The membrane was washed for 3 times with PBST and was then incubated with goat anti-mouse IgG-HRP antibody (diluted at 1:3000, Sigma, United States) for 1 h at RT. The enhanced chemiluminescence chromogenic substrate was quantified by densitometry using ImageJ (NIH, United States). The specific band was visualized with Clarity^TM^ Western ECL Blotting Substrates (Bio-Rad, United States) according to the manufacturer’s protocol. Mouse sera from PBS-treated mice were used as the negative control.

### Immunization and Challenge

A total of 100 female Kunming mice were randomly divided into four groups of 25 mice each. As described in our previous studies (Chen et al., 2016; Zhu et al., 2020), each mouse was injected with 100 μl (1 μg/μl) pVAX-GRA39 plasmid, PBS, empty plasmid pVAX I vector, respectively, and those with no treatment served as blank controls. Blood samples were collected from the tail vein from 25 mice in each group at weeks 0, 2, 4, 6, and stored at −20°C until use.

As described in our previous studies (Chen et al., 2016; Zhang et al., 2018a; Zhu et al., 2020), 2 weeks after the last immunization, 10 mice in all groups were challenged with 10^3^ tachyzoites of the highly virulent *T. gondii* RH strain intraperitoneally and the survival periods were recorded daily until all mice were dead. Meanwhile, the other six mice of each group were orally inoculated with 10 PRU tissue cysts and the mouse brains were removed and homogenized in 1 ml of PBS, and then cysts were morphologically identified and the mean brain cyst loadings were counted under a microscope (40 × objective) on three aliquots of 20 μl, at 4 weeks after the challenge. All samples were counted in triplicate.

Two weeks after the last immunization, a total of nine mice per group were sacrificed and splenocytes were aseptically harvested for flow cytometric analysis (three mice), lymphoproliferation assay (three mice), and cytokine measurements (another three mice).

### Detection of Total *T. gondii* IgG and IgG Subclass Titers

*T. gondii*-specific serum antibody levels were measured by SBA Clonotyping System-HRP Kit (Southern Biotech Co., Ltd, Birmingham, United Kingdom) according to the manufacture’s instruction, as described previously (Chen et al., 2016). Briefly, the 96-well plates were coated with 100 μl (10 μg/ml) TLA diluted in PBS overnight at 4°C. The plates were washed with PBST and blocked PBS containing 1% BSA for 1 h at RT. Mouse serum samples (diluted 1:100 with PBS) were added to the wells and incubated at RT for 1 h. After washing, the wells were incubated with 100 μl of HRP conjugated anti-mouse IgG, anti-mouse IgG1, and IgG2a for 1h. Binding was visualized by incubating with 100 μl substrate solution (pH4.0; 1.05% citrate substrate buffer, 1.5% ABTS, 0.03% H_2_O_2_) for 30 min. The absorbance was measured at 450 nm using an ELISA reader (Bio-TekEL × 800, United States). The highest dilution factor that gives an OD 450 of twice that of the naïve sample at the dilution was designated as the antibody end point titer. All samples were running in triplicate.

### Lymphoproliferation Assay

Splenocyte suspensions from three mice of each group were prepared by pushing the spleens through a wire mesh, and then purified by removing the red blood cells using RBC erythrocyte lysis buffer, and re-suspended in DMEM medium supplemented with 10% fetal calf serum (FCS). In brief, 3 × 10^6^ cells per well were cultured in 96-well plates and cultured with different stimuli, TLA (10 or 5 μg/ml), concanavalin A (ConA; 5 μg/ml; Sigma) as positive control, or medium alone as negative control at 37°C under 5% CO_2_ for 72 h. Then, 10 μl of 3-(4,5-dimethylthylthiazol-2-yl)-2,5-diphenyltetrazolium bromide (MTT, 5 mg/ml, Sigma, Sangon, China) was added to each well and incubated for 4 h. The stimulation index (SI) was calculated as the ratio of the average OD450 value of wells containing antigen-stimulated cells to the average OD450 value of wells containing only cells with medium. All measurements were performed in triplicate.

### Flow Cytometry Analysis

The percentages of CD4+ and CD8+ T lymphocytes were determined by using the flow cytometry analysis, according to our previously described method (Chen et al., 2016; Zhang et al., 2018a). Briefly, splenocyte suspensions were stained with fluorochrome-labeled mAbs including PE-CD3, APC-CD4 and FITC-CD8 (eBioscience) at 4°C for 30 min in the dark, and then fixed with FACScan buffer (PBS containing 1% FCS and 0.1% Sodium azide), and 2% paraformaldehyde. The samples were analyzed for fluorescence profiles on a FACScan flow cytometer (BD Bio-sciences) by SYSTEM II software (Coulter).

### Cytokine Assays

The obtained spleen cells were co-cultured with TLA, ConA (positive control) and medium alone (negative control) in 96-well microtiter plates. Cell-free supernatants were harvested and assayed for IL-2, IL-4 and IL-12p40 at 24 h, for IL-22 activity at 48 h, for IL-10, IL-17A, IL-17F, and IL-23 activity at 72 h, and for gamma interferon (IFN-γ) and IL-12p70 activity at 96 h using commercial ELISA kits according to the manufacturer’s instructions (Biolegend, United States). The analyses were performed in triplicate.

### Quantitative Real-Time PCR

In an effort to determine the pathway mediating the increased production of Th17 and Tc17 responses, the expression of IL-6, TGF-β1, IL-1β, RORγt, RORα, and STAT3 were analyzed by qRT-PCR. Total RNA was isolated from three purified splenocytes of mice in each group by using Trizol reagent (Invitrogen, United States), as per the manufacturer’s instructions. RNAs were dissolved in RNase-free ddH2O (TaKaRa, China) and the cDNA was synthesized using a GoScript^TM^ Reverse Transcription System (Promega, Madison, WI, United States), which were used as templates for quantitative real-time polymerase chain reaction (qRT-PCR). qRT-PCR was performed using the Light Cycler 480 SYBR Green I Master (Roche, Switzerland). The primers used for amplification are listed in Supplementary Materials. qRT-PCR analysis was performed on the Light Cycler 480 (Roche, Switzerland) and data were calculated using the comparative cycle threshold (CT) method (2^–ΔΔ^ ^CT^).

### Statistical Analysis

All statistical analyses were conducted using Graph Pad Prism 5.0 and SPSS17.0 Data Editor (SPSS, Inc, IL, United States). The differences of antibody responses, lympho-proliferation assays, cytokine production, and percentage of CD4+ and CD8+ T cells between all the groups were compared by one-way ANOVA. Survival results are represented by Kaplan–Meier curves and were compared using log rank test. The level of significant difference in comparisons between groups was considered significantly different if *p* < 0.05.

## Results

### Epitope Analysis

**FIGURE 1:**
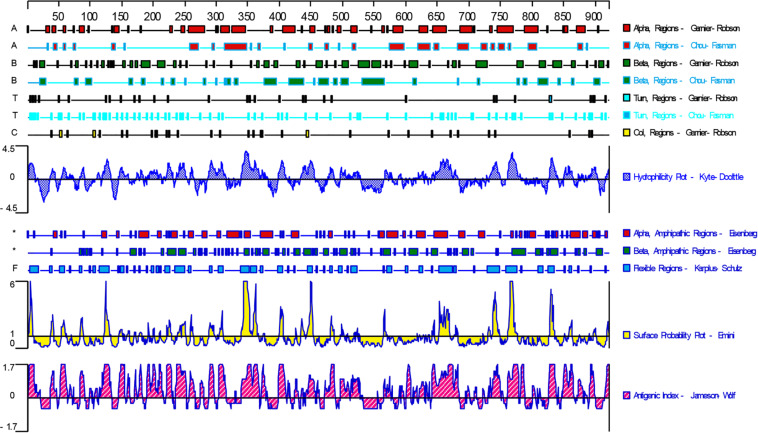
Plot of the DNASTAR-predicted hydrophilicity, flexible regions, antigenic index, and surface probability plot of the linear-B cell epitopes of GRA39.

DNASTAR was used to predict the potential epitopes of the TgGRA39 protein, including surface probability, antigenic index, hydrophilic plot, as well as flexible region. As shown in Figure 1, most regions of TgGRA39 protein were hydrophilicity plots and flexible regions, and TgGRA39 exhibited ideal surface probability and antigenic index, indicating a potentiality of constructing DNA vaccine with it.

### Identification of the GRA39 Gene Expression *in vitro*

**FIGURE 2:**
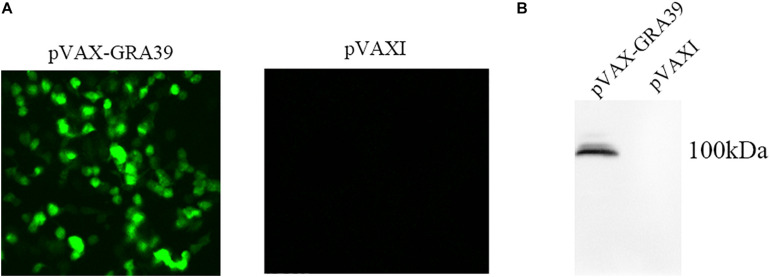
Expression of pVAX-GRA39 in Marc-145 cell. **(A)** Immunofluorescence assay for the recombinant GRA39 protein expressed in Marc-145 cells. **(B)** Western blotting analysis of the expression of GRA39 in Marc-145 cell lysates and empty pVAX I.

Under the fluorescence microscope, specific green fluorescence was observed in the Marc-145 cells transfected with pVAX-GRA39, while no fluorescence was observed in cells transfected with the empty pVAX I (Figure 2A). The western blotting results revealed a single band of about 100 kDa, which was consistent with the expected molecular size, indicating that pVAX-GRA39 was expressed in Marc-145 cells (Figure 2B). On the contrary, no protein band was detected in cells transfected with the empty pVAX1 vector. These results indicated that the GRA39 protein was expressed by pVAX-GRA39 in Marc-145 cells.

### Antibody Detection

**FIGURE 3:**
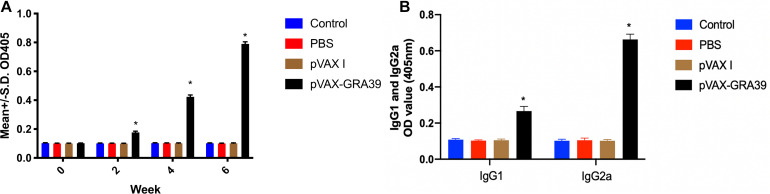
Detection the levels of total IgG, IgG1 and IgG2a antibodies in the sera of Kunming mice. **(A)** Determination of IgG titers induced by DNA immunization at weeks 0, 2, 4, 6. **(B)** Determination of IgG1 and IgG2a titers in the sera of mice two weeks after the last immunization. The results were expressed as means ± SD (*n* = 3) with respect to absorbance at OD450 and statistical differences (*p* < 0.05) are indicated by (*). The bars represented the levels of IgG, IgG1, and IgG2a in the serum of mice.

The antibody titers of IgG and subclasses IgG (IgG1 and IgG2a) in experimental group and the three control groups were detected by standard ELISA. As shown in Figure 3A, the statistically significantly higher levels of endpoint titers of IgG were detected in the sera of mice immunized with pVAX-GRA39, and total antibody levels significantly increased with the continuous immunization, while no increase of antibody titers occurred among the three control groups.

As shown in Figure 3B, the levels of IgG1 and IgG2a endpoint titers were significantly increased in mice immunized with pVAX-GRA39 (*p* < 0.001), in contrast to the three control groups (blank control, PBS, and pVAX I) (*p* < 0.05). In the meanwhile, the ratios of IgG2a/IgG1 were higher in mice immunized with pVAX-GRA39 in comparison with the controls.

### Splenocyte Proliferation

**FIGURE 4:**
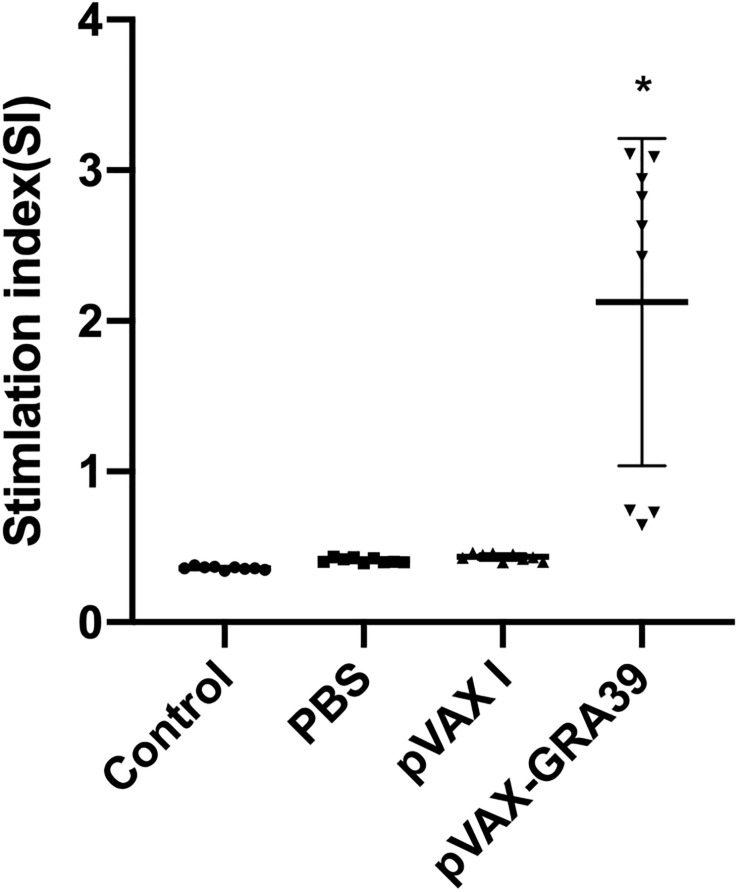
Splenocyte proliferation assay. Two weeks after the last immunization, spleen lymphocytes were collected from vaccinated mice. The proliferative response was evaluated by MTT assay. The results are expressed as the stimulation index (SI) ± SD (*n* = 3). Statistical differences are represented by * (*p* < 0.05).

To analyze the proliferation of splenocytes, the assay was carried out by stimulation of splenocytes with TLA or ConA at two weeks after last immunization. As shown in Figure 4, the proliferative response of lymphocytes was observed on spleen cells, and the proliferation stimulation index (SI) measured at OD450nm in mice immunized with pVAX-GRA39 was significantly higher than in the three controls. However, the three control groups showed no significant difference (*p* > 0.05).

### Flow Cytometry Analysis of Lymphocytes Subpopulations

**FIGURE 5:**
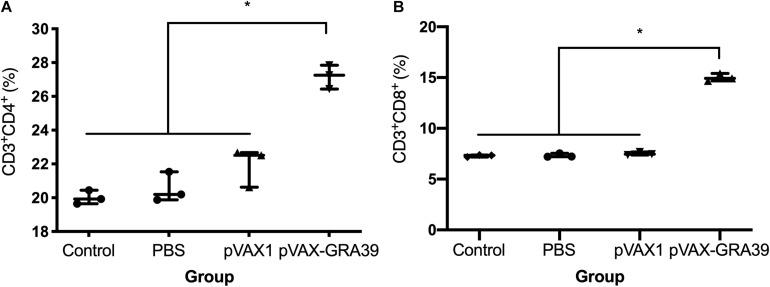
DNA immunization augmented the frequency of antigen-specific T cells in mice. **(A)** Total numbers of CD3+ CD4+ CD8– T lymphocytes per spleen. **(B)** Total numbers of CD3+ CD8+ CD4– T lymphocytes per spleen. Data are mean ± SDs (representative of three experiments). *p < 0.05, compared with the control groups.

As shown in Figure 5, the percentage of CD3+ CD8+ CD4-T lymphocyte subsets (14.78 ± 0.32) in mice immunized with pVAX-GRA39 were much higher than those in the blank (7.34 ± 0.20), PBS (7.41 ± 0.17), pVAX I control (7.40 ± 0.15) group (*p* < 0.05). Also, pVAX-GRA39 group (*p* < 0.05) showed the higher percentage of CD3+ CD4+ CD8-T lymphocyte subsets (26.88 ± 0.35) than that in controls. The ratio of CD8+/CD4+ T cells showed higher level in the group of mice immunized with pVAX-GRA39 in comparison with that of the three control groups.

### Cytokines and Transcription Factors Production

**FIGURE 6:**
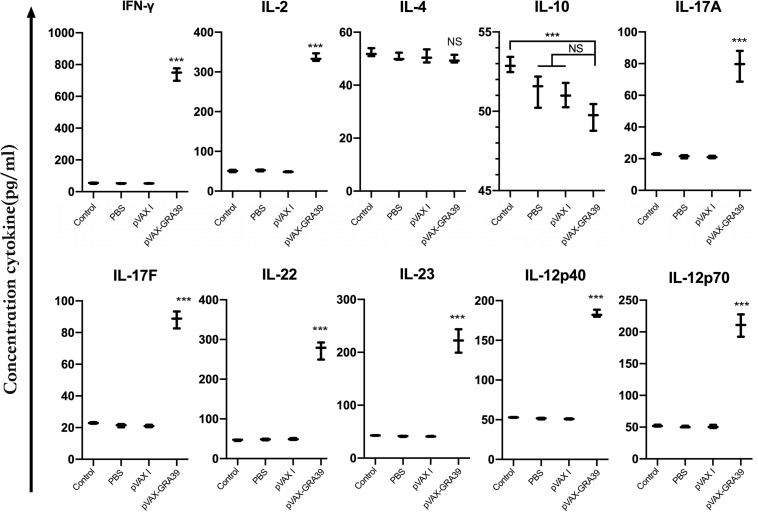
Cytokine production by splenocytes of immunized Kunming mice after stimulation with toxoplasma lysate antigen (TLA). Each bar represents the mean pg/ml (±SE, *n* = 3). ***p < 0.001, compared with the control groups. NS: no significant.

The levels of IL-2, IL-4, IL-10, IL-12p70, IL-12p40, IFN-γ, IL-17A, IL-17F, IL-22, and IL-23 were detected in the mice of experimental group and three controls. As shown in Figure 6, the productions of IFN-γ, IL-2, IL-12p70, and IL-12p40 in the mice immunized with pVAX-GRA39 were significantly higher than that in controls. Besides, increased levels of IL-17A, IL-17F, IL-22, and IL-23 were also detected in mice of experimental group compared to the control groups (*p* < 0.05). The levels of IL-4 and IL-10 cytokines showed no statistically differences to that in three controls (*p* > 0.05).

**FIGURE 7:**
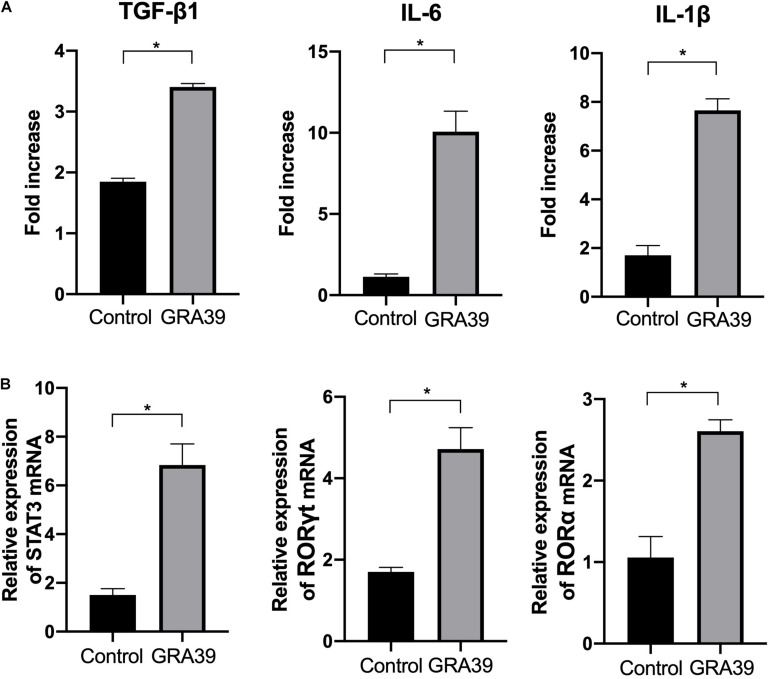
Expression of the cytokines IL-1β, IL-6 and TGF-β1 and transcription factors RORγt, RORα and STAT3. mRNA expression levels of IL-1β, IL-6, TGF-β1, RORγt, RORα and STAT3 were measured in spleens from mice immunized with pVAX-GRA39, and expressed as fold increase over control. **(A)** The mRNA levels of IL-1β, IL-6, TGF-β1; **(B)** The mRNA levels of RORγt, RORα and STAT3. Statistical differences are represented by *(*p* < 0.05).

Expression levels of cytokine gene, IL-6, TGF-β1, and IL-1β, as well as the transcription factors RORγt, RORα, and STAT3 were detected using RT-PCR. We examined the difference in the mRNA level of cytokine genes and transcription factors between control mice and pVAX-GRA39-immunized mice. The expression of IL-6, TGF-β1 and IL-1β, RORγt, RORα, and STAT3 was significantly higher in pVAX-GRA39-immunized mice than in the control group (*p* < 0.05) (Figure 7). These results indicated that increased expression of IL-6, TGF-β1 and IL-1β, RORγt, RORα and STAT3 followed by pVAX1-GRA39 immunization could activate Th17 responses.

### Protective Efficacy of Vaccinated Mice

**FIGURE 8:**
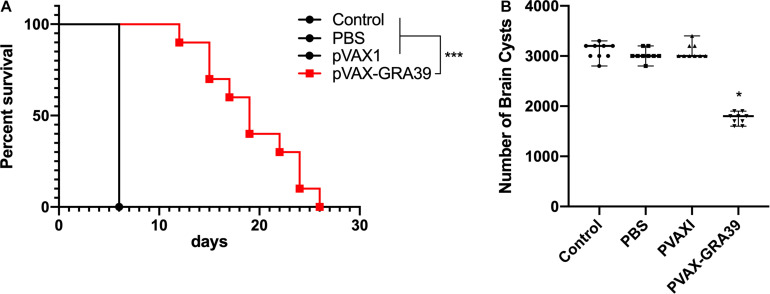
Protective efficacy of mice immunized with pVAX-GRA39 **(A)** Survival curves of Kunming mice after challenge of *T. gondii* RH strain. The mice (10 per group) in all groups were challenged with 1 × 10^3^ tachyzoites of the virulent *T. gondii* RH strain intraperitoneally. Three control groups (PBS, pVAX I and blank control) had 0% survival at day 6. Immunized groups have prolonged survival time of mice. **(B)** Protection against chronic toxoplasmosis in immunized mice two weeks after the last booster immunization. The bars represented the mean cyst burden per mouse brain after oral challenge with a dose of 10 cysts of the Pru strain. Cyst load was counted from whole brain homogenates of mice 4 weeks after challenge. Data are mean ± SDs (representative of three experiments). *p < 0.05, ***p < 0.001 compared with the control groups.

Mortality was observed daily after intraperitoneal challenge with RH strain (1 × 10^3^ tachyzoites) until all the mice of controls and experimental group were dead. As shown in Figure 8A, the mice in the group immunized with pVAX-TgGRA39 significantly prolonged survival time (16.8 ± 3.5) compared to mice in groups of PBS, blank and pVAX I control. While the mice of three control groups died within 6 days after challenge with RH strain, there is no significant difference among the three control groups (*p* > 0.05).

To evaluate the protective efficacy against chronic infection with *T. gondii* PRU strain, tissue cyst loads were examined in brains of the experimental mice and controls at 4 weeks after the third immunization. As shown in Figure 8B, there was a significant reduction (44.5%) in the number of tissue cysts in the brain of the mice immunized with pVAX-TgGRA39 compared to that in three controls (*p* > 0.05).

## Discussion

In recent years, some alternatives have been carried out on vaccines against *T. gondii* in animal models, such as attenuated vaccines (Wang et al., 2017a, 2018), subunit vaccines (Zheng et al., 2013; Ching et al., 2016; Sonaimuthu et al., 2016; Wang et al., 2017b; Gatkowska et al., 2018), exosome vaccines (Beauvillain et al., 2009; Li and Zhou, 2018), DNA vaccines (Zhang et al., 2015b), and other types of vaccines (Lee et al., 2018; Zhang et al., 2018a). Due to the their low production cost and thermal stability, as well as their ability to induce cellular and humoral immune responses, DNA vaccines have become promising method used for defending against *T. gondii* infection in animal models (Cao et al., 2015; Li and Zhou, 2018). Prior to evaluating the potentiality of DNA vaccine candidate, bioinformatics has been widely used as an experimental methodology, and also been used to predict gene structures, functions, and epitopes, or to design new vaccine candidates. Among those epitopes, some molecules in the studies of *T. gondii* DNA vaccines have been predicted by the immunogenicity of some molecules, including SAG4 (Zhou and Wang, 2017), ROP21 (Zhang et al., 2018c), TgDOC2C (Zhang et al., 2018b), GRA24 (Zheng et al., 2019), etc. In the present study, the bioinformatics analysis of TgGRA39 protein showed that most regions of the TgGRA39 protein hydrophilicity plots and flexible regions, suggesting an excellent antigenic index and surface probability, which is indicative of a promising vaccine candidate.

In previous studies, some DNA vaccine candidates encoding GRA protein of *T. gondii*, such as GRA6, GRA7, GRA2, GRA15, GRA16, GRA24, and GRA25, have demonstrated some efficacy against *T. gondii* infection, with their ability of eliciting immunity, particularly cellular immune responses, which is critical for defending against acute and chronic toxoplasmosis in animal models (Chen et al., 2015; Hu et al., 2017; Xu et al., 2019; Zheng et al., 2019). In the present study, we constructed a DNA vaccine expressing TgGRA39, and evaluated its immunogenicity and protective efficacy against infection with the highly virulent *T. gondii* RH strain and the chronic *T. gondii* PRU strain. Our results revealed that immunization with pVAX-GRA39 can evoke specific humoral and cellular immune responses, resulting in effective protective immunity against acute and chronic toxoplasmosis in Kunming mice.

Th1-type immune response is considered to play a crucial role in resistance against *T. gondii* infection (Gigley et al., 2009). IFN-γ, the cytokine of the Th1-type lymphocytes, is confirmed to induce inflammatory response and thus to control *T. gondii* load during early stages of infection (Silva et al., 2009). IL-2, another important Th1-biased cytokine, could regulate the proliferation and activities of CTLs, which are important for resistance against *T. gondii* infection (Karna et al., 2009). IL-12 (IL-12p70) is the determinant of Th1 cells immune response, which could promote the production of Th1-biased cytokine, such as IFN-γ, effectively (LaRosa et al., 2008). Besides, IL-12p40 could also promote the proliferation of T cells including memory T cells and the generation of IFN-γ (Karna et al., 2009). In the present study, significantly increased levels of IFN-γ, IL-12p70, IL-12p40, and IL-2 were detected in the group of mice immunized with pVAX-GRA39, suggesting that Th1-type mediated immunity was elicited by pVAX-GRA39 injection in mice successfully, which are essential for the prolonged survival and reduced brain cysts in the immunized mice. In contrast, as marker cytokines for Th2 cells, IL-4, and IL-10 secreted from the group of mice immunized with pVAX-GRA39 was not significantly higher than that in the control groups, which is an indicative of a more pronounced Th1 response than a Th2 response. However, the absence of a IL-4 and IL-10 response followed by DNA immunization with pVAX-GRA39 may be harmful for the *T. gondii* infective process, which is ascribed to their regulatory role of an extreme Th1 response, involved in limiting the inflammation and inhibiting CD4+ T cell-mediated severe immunopathology (Dupont et al., 2012).

Apart from Th1 cytokines, Th17-mediated protective cellular immunity induced by vaccine have been demonstrated to play a critical role in defending against some pathogens, including *Mycobacterium tuberculosis*, *Helicobacter pylori*, *Pseudomonas aeruginosa* (Khader et al., 2007; Priebe et al., 2008; DeLyria et al., 2009). Th17 cells, this new lineage of T helper cells, were recognized for producing pro-inflammatory cytokines, including IL-17, IL-22, and IL-23 (Ghosh et al., 2013; Sacramento et al., 2014). Among these cytokines, IL-17A produced by CD4+ Th17 cells was found to play a protective role against the intracellular pathogen *Trypanosoma cruzi*, *Leishmania donovani*, and *Leishmania infantum* (Banerjee et al., 2016; Cai et al., 2016). However, evaluation of Th17 responses during immunization trials against *T. gondii* received less attention compared to Th1 and Th2 responses. In our study, after DNA immunization with pVAX-GRA39, a significant increase of IL-17A, IL-17F, IL-22, and IL-23 was detected, suggesting the activated Th17 responses, which is in accordance with some previous studies on immune responses induced by *L. donovani* and *M. tuberculosis* vaccines (Khader et al., 2007; Priebe et al., 2008; DeLyria et al., 2009).

Currently, IL-6, TGF-β1, IL-1β have been considered to be Th17-inducing cytokines, which could prime naive T cell (Th0) differentiation toward Th1/Th2/Treg/Th17 phenotype, and play important roles in the differentiation, proliferation, and maintenance of Th17 cells (Mills, 2008; Korn et al., 2009). Both IL-6 and TGF-β1 could prime naïve CD8+ T cells toward Tc17 cells, thereby, express hallmark molecules, such as retinoic acid receptor-related orphan receptor (ROR)gammat, RORalpha and IL-21 (Yen et al., 2009; Srenathan et al., 2016). Also, the development of Tc17 cell is indispensably dependent on STAT3 and can be augmented by increased expression of RORγt (Huber et al., 2009). In the present study, a significant increase in the expression level of IL-6, TGF-β1, IL-1β, and the transcription factor factors RORγt, RORα, and STAT3 was observed in mice immunized with pVAX-GRA39 compared to mice in the control group, indicating that Tc17 cells responses were activated in CD8+ T cells through RORγt and STAT3 following DNA immunization with pVAX-GRA39. Our findings indicated that pVAX-GRA39 was capable of evoking Th17 and Tc17 differentiation, resulting in a pro-inflammatory protective immunity. A distinct subset of IL–17–producing CD8+ T cells had been identified and designated as Tc17 cells, which are recognized as playing a protective role in host against vaccinia and influenza virus infections (Yeh et al., 2010; Hamada et al., 2013), and are also indispensable for protective immunity against fungal pneumonia (Nanjappa et al., 2015). Recently, it has been established that Tc17 cells could secrete IL-17F and IL-22, and express IL-23R, along with Th17 lineage-specific transcription factors RORγ and RORα (Stritesky et al., 2008; Glosson et al., 2014). These encouraging results in combination with our findings have emphasized that Th17- and Tc17-associated responses induced by DNA immunization with pVAX-GRA39 may play an important role in host defending against *T. gondii* infection.

During the *T. gondii* invasion, T-cell-mediated immunity is also dominant in the process of host immune response for mediating resistance to *T. gondii* infection (Jongert et al., 2010; Zhang et al., 2015a). Especially, CD8+ T cells are specialized cytotoxic T lymphocyte cell that mediate lysis of *T. gondii* by the production of IFN-γ or perforin-mediated cytolysis, in synergy with CD4+ T cells (Gigley et al., 2009; Dupont et al., 2012). The data in the present study was consistent with some previous reports, underlying the protective effects of those vaccines (Xu et al., 2014; Zhang et al., 2014), with the activated proliferative response of lymphocytes and significant increase of both CD8+ and CD4+ T cells in immunized mice. These increased levels of CD4+ and CD8+ T cells further emphasized that T cell-associated immune responses hold a position in resistance against *T. gondii* infection followed by pVAX-GRA39 immunization.

Humoral responses were well known to play an important role in immunity against *T. gondii*, especially, specific antibodies can attached the parasite to the host cell receptors or to the complement molecular, involving in antibody-mediated activation of the classical pathways of complement (Sayles et al., 2000). Augmented levels of IgG were detected in immunized group by ELISA assay, suggesting that DNA immunization pVAX-GRA39 activated humoral immune response effectively, which may be beneficial to opsonize the parasite for phagocytosis and block invasion (Sayles et al., 2000). In addition, pVAX-GRA39 immunization has induced a significant high ratio of IgG2a to IgG1 titers, a characteristic of the Th1-type response in contrast to three control groups.

Similar with previous studies (Chen et al., 2013, 2017; Gao et al., 2018), DNA immunization with a single antigen of TgGRA39 could only induce partial protective immunity in Kunming mice, due to its limited lymphocyte binding sites, leading to a difficulty of mounting an efficient immune response against *T. gondii* (Donnelly et al., 2005). Although this single DNA immunization with pVAX-GRA39 has elicited similar or different effective immunity in comparison with some other single DNA vaccine candidates, such as TgGRA24, TgGRA25 and TgGRA16 (Hu et al., 2017; Xu et al., 2019; Zheng et al., 2019), this DNA immunization has evoked Th17 responses, which are commonly directly correlated with mucosa protection, and also considered to be beneficial in limiting the host infection via the gastrointestinal tract (Gaffen and Moutsopoulos, 2020). However, in those previous studies on *T. gondii* vaccines, detection of Th17 responses was rarely found. So, these effective responses induced by DNA immunization with pVAX-GRA39 could limit the infection with *T. gondii* via the gastrointestinal tract, leading to the reduction of brain cysts and prolonged survival time in mice models in combination with activated Th1-responses. In addition, the challenge dose of *T. gondii* has been recognized as an important impact factor in analyzing the immune protective effect (Li and Zhou, 2018). Especially, the vaccine could elicit the protective immunity, but it is unable to fight too high a dosage of the lethal *T. gondii* parasite strain RH, resulting in failing observation of a longer survival time due to high the dosage (Yan et al., 2012). So, the low-virulence strain PRU may be more suitable to study survival after challenge, and further study can be explored to 80 cysts of strain PRU per mouse were administered intragastrically in order to study survival after challenge, according to some previous studies (Yan et al., 2012). Moreover, further studies should also evaluate the role of limited lymphocyte binding sites of TgGRA39 by adding immunotargeting molecules to the antigen, and also the immunity against *T. gondii* infection with TgGRA39-based multiantigenic vaccine, and even electroporation can be further used to enhance the protective immune responses, associated with the increased duration of survival and/or fewer cysts.

In conclusion, our work demonstrated that TgGRA39 is a DNA vaccine candidate in Kunming mice model, with ability of eliciting humoral immune responses and Th1-biased, Th17 and Tc17 responses. This potential vaccine candidate can prolong the survival time in mice infected with RH strain and reduce the number of cysts in mice infected with PRU strain, providing a foundation for further multi-epitope vaccine design based on TgGRA39.

## Data Availability Statement

The original contributions presented in the study are included in the article/Supplementary Material, further inquiries can be directed to the corresponding authors.

## Ethics Statement

The animal study was reviewed and approved by the ethical committee of Ningbo University (permission: SYXK(ZHE)2013-0190).

## Author Contributions

JC, CZ, and YZ were involved in the final development of the project and manuscript preparation. YX analyzed the data. YZ and LH performed most of experiments. All authors contributed to the article and approved the submitted version.

## Conflict of Interest

The authors declare that the research was conducted in the absence of any commercial or financial relationships that could be construed as a potential conflict of interest.
