# Supplementary material for: Immunization With a DNA Vaccine Encoding the Toxoplasma gondii’ s GRA39 Prolongs Survival and Reduce Brain Cyst Formation in a Murine Model
Source: Front Microbiol. 2021 Apr 28;12:630682. doi: 10.3389/fmicb.2021.630682 (PMC8113873; doi:10.3389/fmicb.2021.630682)
Supplement: Supplementary file 1 [file Table_1.DOCX]

**Supplementary Materials**

**Table S1 Primer sequences.**

| **Gene** | **Sequence (5′→3′)** |
| --- | --- |
| IL-6 | Sense: 5’-ACACGCTGTCCTTACACTGG -3’  Anti-sense: 5’-CTTCTGATTGCCCTCTGGGG -3’ |
| TGF-β1 | Sense: 5’-ACACGCTGTCCTTACACTGG -3’  Anti-sense: 5’- CTTCTGATTGCCCTCTGGGG-3’ |
| IL-1β | Sense: 5’ – CACCAGCATCTTTTCCAACC -3’  Anti-sense: 5’- AAGGCCGACTCTCCTACACA -3’ |
| RORγt | Sense: 5’- CGAGCACAGAATCGCTTCA -3’  Anti-sense: 5’- CTCGCTTCGGCAGCACATAT -3’ |
| RORα | Sense: 5’- ATGGCTTTACCATTGCGTGTT -3’  Anti-sense: 5’- TTAATTCTGCGTCGTTACGGT -3’ |
| STAT3 | Sense: 5’- CCGACGAGTGGAGAAATC -3’  Anti-sense: 5’- AGCAGGATGTGGAGGTAG -3’ |
